# Correspondence

**Published:** 1881-02

**Authors:** G. Watt

**Affiliations:** Xenia, O.


### CORRESPONDENCE.

Dr. J. Taft, Editor of Dental Register.

Dear Sir — Week before last, as it seems in the fond freshness of friendship's memory, though it was in December of 1835, you and I, as equal partners, re-roofed the stable of the principal of Taylor's Academy. There was nothing of discord as to who should carry boards, who drive nails, or who fall off the scaffold. In all the years, up to date, we have been found at work, with similarity of aim and effort, and the accord of the first combined contract proved typical of all that has followed. If we have been of use to our fellow men or to our profession, it is probable much of our usefulness has been due to the fact that we were, in early life, thrown together, and that each has been a stimulant to the other in our endeavors to climb the hill of science. How often have I, weary, worn and discouraged, laid down my pen, only to take it up again, when aroused and encouraged by a word or look from you. With tastes so dissimilar, it is strange that we embarked on the same channel of effort. But strange as it is, you well know that this occurred, not because we designed it so, but in obedience to the Yankee sawmill power known as the force of circumstances.

How little was known by the dental profession when we came into it! and how little we know yet! The therapeutic agents employed by dentists then were all, might I say, used empirically. Of their nature, composition, and *modus operandi*, but little was known. The direct chemical agents producing decay of the teeth were not known; and there was little, if any, discriminating thought in regard to them. Acids, in general, were spoken of as causing caries of the teeth, in such a way as to suggest that carbolic acid had as much to do with it as hydrochloric. Men eminent in the profession were holding that there is but one kind of caries, the difference in appearance being due to the age of the decay, character of the teeth, etc. The welding properties of gold were unknown and unrecognized, except by a very few. You well remember that, as late as 1856, you and I were insulted, in a meeting of the American Dental Convention, for claiming that gold can be welded at common temperatures. Said a member, "If it be true that gold is welded at ordinary temperatures, or at *any* temperature, a new fact in the science of chemistry has been, this day, announced; but, Mr. President, the world has heard of young upstarts before." But the thought lived; and we did, too.

In thinking of the darkness that has been dispelled, we find pleasure; and the thought fills us with hope for the future. Though you and I have done more work in the past than we can expect to do in the future, yet the work will go on. Our own students would see to that, if there were no thinkers beside. But when we look over the field of our profession, we have great reason to take courage; for though much work is yet to be done, there are many workers, and the moral momentum acquired by the effort of the past generation, we trust, will greatly aid in making progress in the generation to come.

Do you ever stop to think how lonely we should feel had it not been that new hands were daily added to the list of laborers?

Many, whose names were as watchwords among the workers, now lie low in the dust; but we have the consolation that

> "Dust thou art — to dust returnest,
> Was not spoken of the soul."

and this thought, as well that "their works do follow them," in the thoughts and efforts of others whose energies have been aroused and quickened by witnessing the earnestness of their struggles in behalf of professional advancement. We feel that Time, the harvester, must have laid aside his old hour-glass and scythe, and seized a gold repeater, and mounted a self-raker and binder, when we think of Harris, Piggott, J. D. White, Jones, White, and McCurdy, C. C. Allen, Edward and Joseph Taylor, A. M. Leslie, McQuillen, Barker, H. R. Smith, J. B. Smith, Thos. Wood, Mendenhall, Wildman, Austen, Arthur, Solyman, Brown, Flagg, Westcott, Blakesley, Peebles, Shotwell, and many others. These worked with us, and we with them; and to me it seems passing strange, that so many of these have gone before, while I am still left to toil on as I may. With you, who have never relaxed, or left your post, the feeling may not be so peculiar, for you have been ever busy with them, and as it were, near them, when they dropped by the way. But having been for a time on the shelf, their departure, when considered from a professional standpoint, is, to me, more like the announcement of a single news item. The question arises, and refuses to lie quiet, Can we spare all these men and their labors from our profession, and still make progress? But new men, new thinkers, new thoughts, have responded to the order of the genius of our profession, "Go, work to-day in my vineyard." As already said, the work will go on, and the workers will be found as demanded.

But, are you not well aware that many claiming connection with our profession think that our work is already done, — our progress already made? When you ask such to write an article for the *Register* do they not respond, in substance, O! there is nothing to write about; every subject is exhausted? In view of this state of feeling, which is far more prevalent than any but an editor of a professional journal would suppose, it may be well to suggest or indicate some directions in which progress is demanded :—

And right here the thought takes the direction of dental hygiene. How shall the mother's health be so promoted that she may be able, from the normal richness of her own blood, to impart health and vigor to her infant, to such extent that it can develope strong, hard, healthy, well formed dental organs? The periods of gestation and lactation, call for more attention to the mother than to the child; but our profession, in its present state of attainments, is not able to give the fond mother the attention and advice she needs for these periods. Then, that the babe's health shall not suffer in weaning, in such way as to deteriorate the teeth; and so on, through first dentition, through second dentition, through the changes of puberty. Ask yourselves, each one, editor, reader, teacher, pupil, do you know half as much as you ought to know of these things?—half as much as you wish to know?

Again, painful operations will be sometimes necessary for a time, at least. How shall they be performed? and with what instruments? There is much room for improvement in this direction. And suppose an anæsthetic is to be used. How soon can we get the profession and the public to understand that unconsciousness, and especially delirium are not essential to anæsthesia? And how soon can we succeed satisfactorily with an anæsthetic agent that shall introduce no foreign, at least, no objectionable substance into the blood, that shall leave the patient conscious throughout the process, while the functions of life are undisturbed, except the ability to feel pain?

And, further, how soon shall we have a material for filling decayed teeth free from the objections that lie against gold, tin, the plastic agents, and especially the infernal amalgams? There is room for thought in this direction.

And who shall teach us all to control toothache from reflex action? Why must prospective young mothers suffer the untold agonies of odontalgia, even when their teeth are all sound? Why, indeed! except that we are too ignorant to relieve them.

But space will not permit further enumeration. In short, there is no direction in which we cannot find darkness that must be dispelled. Let all, then, awake from their lethergy and get earnestly to work, and the race will be blessed as a result.

And now I must close. Again, in the years of our silver gray, we both sail on the sea of journalism. On account of editorial responsibility elsewhere, I shall probably write less frequently for the Register than if the facts were otherwise ; yet shall the Register ever have a warm place in my memory, and I shall ever rejoice in its prosperity. Though the oldest of us all, may it ever have the vigor of youth.

G. Watt.

Xenia, O., Dec. 25, 1880.

